# Enhancing the light absorbance of polymer solar cells by introducing pulsed laser-deposited CuIn_0.8_Ga_0.2_Se_2_ nanoparticles

**DOI:** 10.1186/1556-276X-9-308

**Published:** 2014-06-17

**Authors:** Yu Zhao, Hui Li, Xu-Jun Liu, Lei-Lei Guan, Yan-Li Li, Jian Sun, Zhi-Feng Ying, Jia-Da Wu, Ning Xu

**Affiliations:** 1 Department of Optical Science and Engineering, Shanghai Ultra-Precision Optical Manufacturing Engineering Center, Fudan University, Shanghai 200433, People’s Republic of China; 2 Department of Physics, Shanghai Electric Power University, Shanghai 201300, China

**Keywords:** CuIn_0.8_Ga_0.2_Se_2_ nanoparticles, P3HT:PCBM, Pulsed laser deposition, Absorption, Polymer solar cells, Photoluminescence

## Abstract

Evenly separated crystalline CuIn_0.8_Ga_0.2_Se_2_ (CIGS) nanoparticles are deposited on ITO-glass substrate by pulsed laser deposition. Such CIGS layers are introduced between conjugated polymer layers and ITO-glass substrates for enhancing light absorbance of polymer solar cells. The P3HT:PCBM absorbance between 300 and 650 nm is enhanced obviously due to the introduction of CIGS nanoparticles. The current density-voltage curves of a P3HT:PCBM/CIGS solar cell demonstrate that the short-circuit current density is improved from 0.77 to 1.20 mA/cm^2^. The photoluminescence spectra show that the excitons in the polymer are obviously quenched, suggesting that the charge transfer between the P3HT:PCBM and CIGS occurred. The results reveal that the CIGS nanoparticles may exhibit the localized surface plasmon resonance effect just as metallic nanostructures.

**PACS:**

61.46. + w; 61.41.e; 81.15.Fg; 81.07.b

## Background

In recent years, polymer-fullerene-based bulk heterojunction (BHJ) solar cells aroused the interest of researchers and manufacturers due to their low cost, large areas, and flexibility [1-3]. However, compared with crystalline silicon cells, the efficiency of polymer-fullerene BHJ solar cells is still much lower. One of the main factors limiting their efficiency is the low light absorption and low charge carrier mobility of polymer absorbers. For example, the poly(3-hexylthiophene) (P3HT) mixed with phenyl-C61-butyric acid methyl ester (PCBM), a commonly used conjugated polymer absorber in polymer-fullerene BHJ solar cells, has quite a large bandgap of about 2.1 eV, determining that it can only absorb the incident light whose wavelength is shorter than 590 nm. Moreover, the carrier mobility of P3HT is only in magnitude of 10^-3^cm^2^V^-1^s^-1^, which will lead to severe carrier recombination in transport through the thick P3HT:PCBM active layer. So, the practical thickness of the P3HT:PCBM active layer is commonly limited to be about 200 nm, and almost half of incident light can not be absorbed by the active layer. In order to resolve these problems, various inorganic materials with shorter bandgaps or higher carrier mobility including CdS, CdSe, and CuInS_2_ were introduced into organic solar cells to fabricate hybrid solar cells to enhance their light absorption and carrier mobility [4-7]. For example, nanoparticles of CuInS_2_ have been embedded into conjugated polymer blends to fabricate hybrid solar cells [7]. Compared with these inorganic materials, CuInSe_2_ has a lower energy gap (1.02 eV), which leads to a considerably high absorption coefficient (about 10^5^ cm^-1^), even higher than that of CuInS_2_. If different element ratios of Ga are added into CuInSe_2_, the bandgap and energy level of the formed CuIn_*x*_Ga_1- *x*_Se_2_ (CIGS) can be adjusted to match better with those of ITO electrodes and organic materials to achieve higher open voltage [8]. Furthermore, the CIGS has good conductivity, and its conductivity type depends on its stoichiometry, which can easily be varied in the synthesis processes according to the design of the solar cell. This is beneficial to fabricate the hybrid solar cells with different structures. Therefore, the CIGS is potential for use as inorganic absorbers in the hybrid solar cells.

So far, several deposition and post-treatment techniques, such as thermal co-evaporation, sputtering, electrodeposition, and selenization of prefabricated metallic layers, have been tried to achieve the requirements for CIGS syntheses [9-12]. The difficulties to control the stoichiometry of the CIGS thin films make these processes very complicated and much expensive. As one of the alternative techniques, pulsed laser deposition (PLD) is a convenient, economical, and effective method to deposit multi-component films because of its congruent ablation proceedings [13,14]. In this article, a YAG:Nd laser was used in PLD to deposit CuIn_0.8_Ga_0.2_Se_2_ nanoparticles on ITO-glass substrates. The CIGS nanoparticles deposited at 400°C were introduced between the conjugated polymer layers and ITO electrodes in the photovoltaic structures of polymer solar cells to improve their light absorption and current density-voltage performance. The mechanism of the enhancement of the light absorption and photoelectric conversion of the photovoltaic structure was investigated.

## Methods

Conventional polymer solar cells were fabricated in this following procedure: Cleaned ITO-glass substrates were spin-coated by highly conducting poly(3,4-ethylenedioxythiophene)/poly(styrene sulfonic acid) (PEDOT:PSS; Clevios 4083, Heraeus, Hanau, Germany) at 2,000 r/m for 40 s. After being annealed on a hot plate at 150°C for 10 min in order to remove moisture, the samples were spin-coated by a mixed solution of P3HT:PCBM with concentrations of 15 and 12 mg⋅ml^-1^ in dichlorobenzene at 2,000 r/m for 40 s. Then, the samples were annealed on a hot plate at 150°C for 20 min to remove dichlorobenzene. The whole process was completed in a nitrogen glove box. Finally, Al thin films with a thickness of 150 nm as the cathodes were deposited onto the above layers by magnetron sputtering method through a shadow mask, resulting in active device areas of 7 mm^2^. The completed photovoltaic structure of ITO/PEDOT:PSS/P3HT:PCBM/Al was annealed at 150°C for 30 min in the nitrogen glove box.

**Figure 1:**
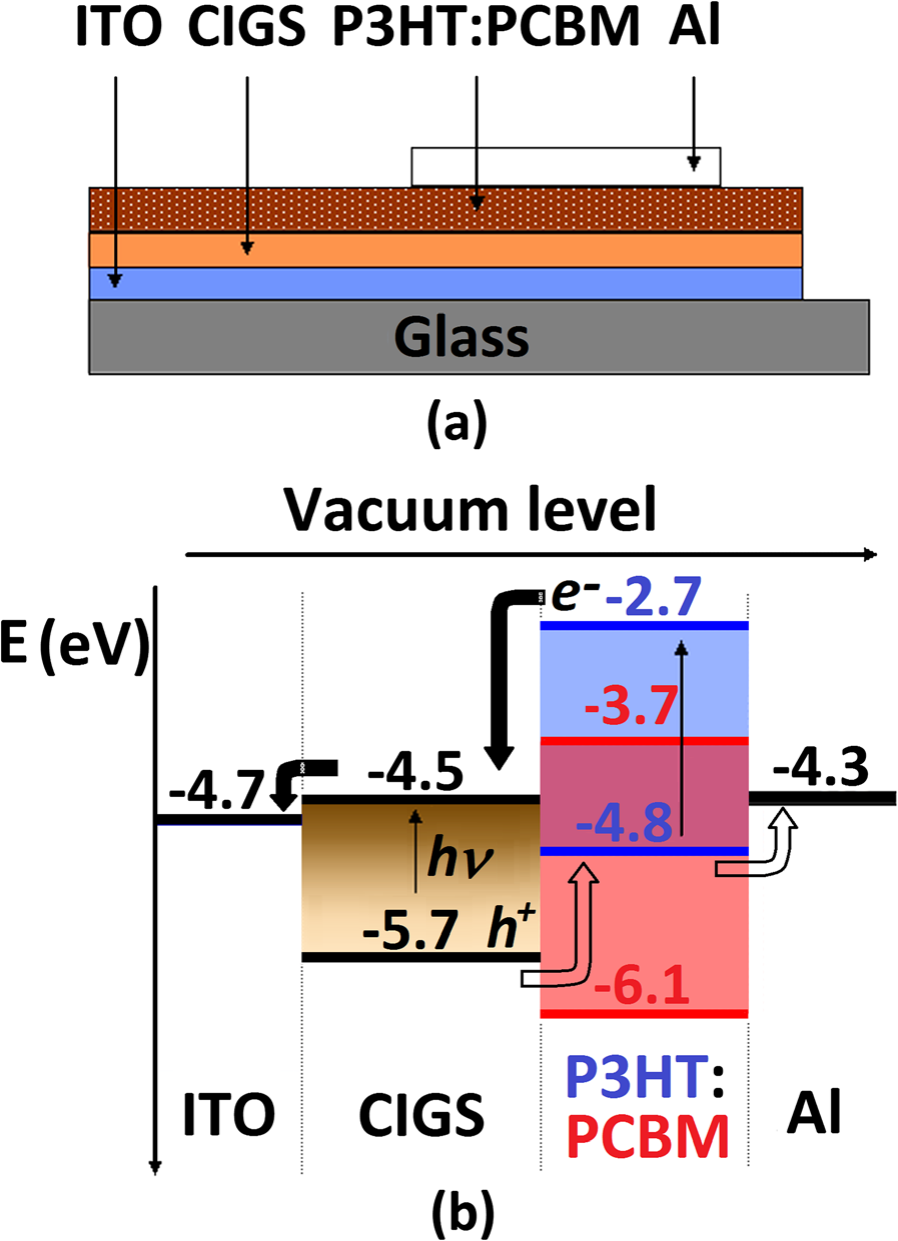
**Layout of a CIGS-based hybrid solar cell and its schematic energy level diagram. (a)** Layout of the CIGS-based hybrid solar cell with the structure of ITO/CIGS/P3HT:PCBM/Al. **(b)** Schematic energy level diagram for the above structure (with energy levels in electron voltage relative to vacuum).

The preparation process of the CIGS-based polymer solar cells with the structure of ITO/CIGS/P3HT:PCBM/Al (shown in Figure 1a) was similar with that of the conventional polymer solar cell except that the ITO-glass substrates were covered by the layers of the CIGS nanoparticles deposited by PLD replacing the conventional PEDOT:PSS layers. The experimental setup of PLD consists of a Nd:YAG laser with a wavelength of 532 nm, a pulse duration of 5 ns, a deposition chamber with a rotating multi-target, and a base pressure of 1 × 10^-6^ Torr. The laser beam was arranged to be incident at 45° on a target surface through a quartz window. The laser energy and repetition rate were 50 mJ and 10 Hz, respectively. The CIGS nanoparticles were deposited using a hot-pressed CuIn_0.8_Ga_0.2_Se_2_ target at a substrate temperature of 400°C for 3 min.

The surface and cross-sectional morphology of the CIGS layers and CIGS/P3HT:PCBM bilayer was characterized by scanning electron microscopy (SEM) (XL30FEG, Philips, Amsterdam, Netherlands). The composition of the CIGS nanoparticles was analyzed by energy dispersive spectroscopy (EDS) fitted on the SEM. The crystallinity of the CIGS layers was examined by X-ray diffraction (XRD) (D/MAX-IIA, Rigaku, Tokyo, Japan) using the Cu Kα radiation. The UV-vis absorption spectroscopy of the P3HT:PCBM blend monolayer and CIGS/P3HT:PCBM bilayer was detected by an ultraviolet-visible spectrophotometer (U-3000, Hitachi, Tokyo, Japan). The current density-voltage (*J*-*V*) characteristics of the unencapsulated samples were tested in air by using a Keithley 2400 SourceMeter (Cleveland, Ohio, USA) under air mass (AM) 1.5 global solar condition at 100 mW/cm^2^. The photoluminescence (PL) of the P3HT:PCBM blend monolayer and CIGS/P3HT:PCBM bilayer was measured at room temperature using a 325-nm He-Cd laser as the exciting light source. The PL spectra were detected by collecting the luminescence with a spectrometer (Spectra Pro 500i, Acton Research, Trenton, NJ, USA) and recorded by an intensified charge-coupled device (ICCD; iStar DH720, Andor Technology, Belfast, UK) installed on the exit port of the spectrometer.

## Results and discussion

**Figure 2:**
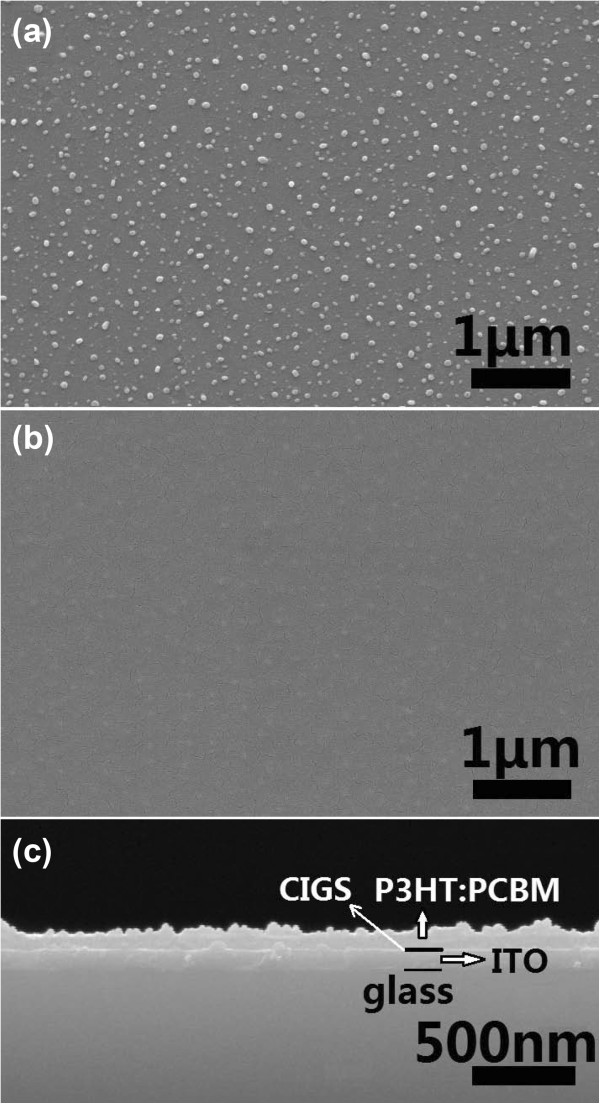
**SEM images. (a)** The surface of a CIGS layer, **(b)** the surface of a CIGS/P3HT:PCBM bilayer, and **(c)** the cross-section of the CIGS/P3HT:PCBM bilayer. The CIGS layers were deposited at a substrate temperature of 400°C for 3 min.

Figure 2a,b,c shows the SEM images of the surfaces of a CIGS layer and a CIGS/P3HT:PCBM bilayer and the cross-section of the CIGS/P3HT:PCBM bilayer. As seen in Figure 2a, there are evenly separated nanoparticles with sizes of 20 to 70 nm and a distribution density of about 7 × 10^9^ cm^-2^ on the surface of the ITO-glass substrate. Figure 2b shows that the CIGS nanoparticles under the spin-coated P3HT:PCBM layer can still be perceived. In Figure 2c, almost no voids can be observed between the ITO thin film, CIGS nanoparticles, and the above polymer layer. The closely contacting interface between them is vital for the separation of electron-hole pairs and the transportation of electrons or holes, which are important for the hybrid solar cells to obtain high performance [15].

**Figure 3:**
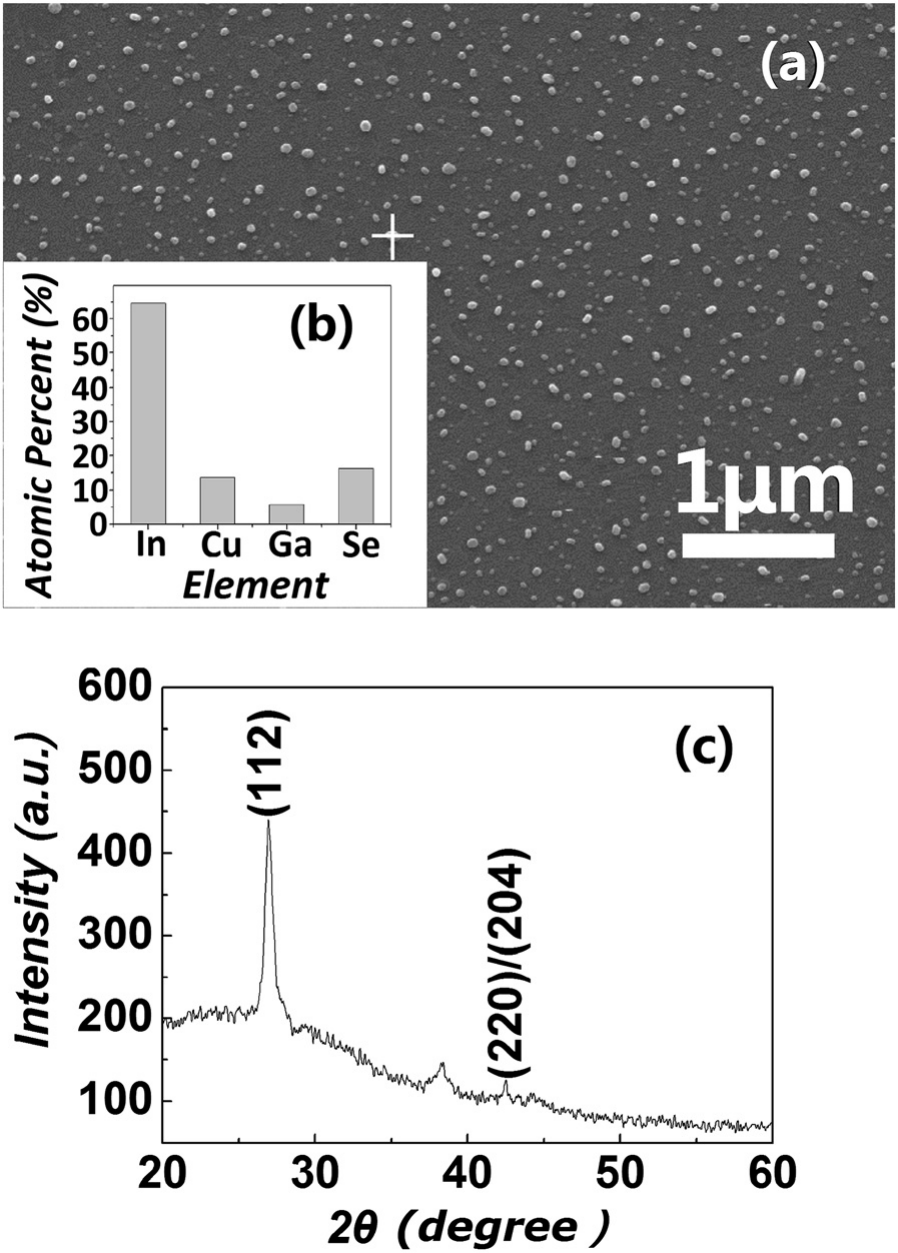
**Surface SEM image, EDS spectrum, and XRD pattern of a CIGS layer.** The CIGS layer was deposited at a substrate temperature of 400°C for 3 min. **(a)** The surface SEM images of the CIGS layer, **(b)** the analysis results of the EDS spectrum of the CIGS nanoparticle at the position marked by a white cross in **(a)**, and **(c)** the XRD pattern of the CIGS layer shown in **(a)**.

**Figure 4:**
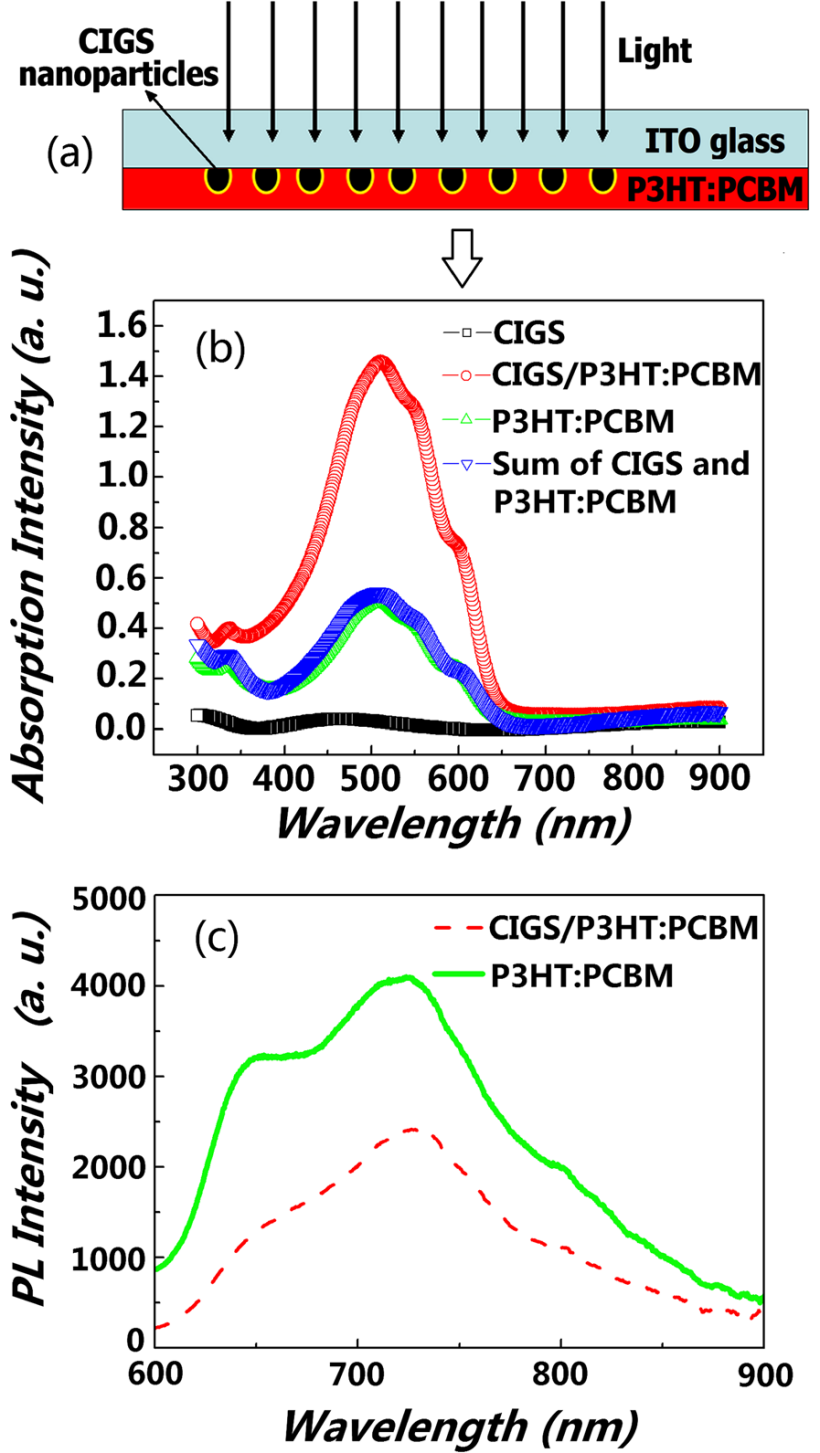
**Schematic of LSPR light trapping, UV-vis absorption spectra, and PL spectra. (a)** Schematic of LSPR light trapping for a hybrid system of ITO/CIGS/P3HT:PCBM in which the CIGS nanoparticles are embedded between the ITO substrate and P3HT:PCBM photoactive layer. **(b)** The UV-vis absorption spectra of ITO/CIGS, ITO/P3HT:PCBM, and ITO/CIGS/P3HT:PCBM. **(c)** The PL spectra of ITO/P3HT:PCBM and ITO/CIGS/P3HT:PCBM.

In order to know the composition of the as-deposited nanoparticles, EDS was carried out at the places with and without the as-deposited nanoparticles. Figure 3b gives the EDS analysis result of an as-deposited nanoparticle shown in Figure 3a (marked by a white cross). The elements Sn, C, and O are not included in the EDS analyses for they come from the ITO thin film and because they were exposed to air for a long time. In Figure 3b, the percentages of In, Cu, Ga, and Se are about 64.57%, 13.47%, 5.68%, and 16.28%, respectively. Due to the In contribution from the ITO film, the detected In content is far more than the stoichiometry of the CIGS. Because the EDS is only a semi-quantitative analysis tool, its analysis results are usually of some deviation from the actual situation. At the places without nanoparticles, the elements Cu, Ga, and Se are below the detection limit of the EDS device. The co-existence of In, Cu, Ga, and Se only in the nanoparticles indicates that the as-deposited CIGS layer is composed of scattered CIGS nanoparticles. To further understand the structure of the as-deposited CIGS nanoparticles, XRD was also measured to examine the crystallinity of the CIGS layer. Figure 3c shows the XRD pattern of the as-deposited CIGS layer. In Figure 3c,the distinct (112) peak of the chalcopyrite phases of CIGS can be characterized [12], and the average grain size calculated by the Debye-Scherrer formula is 28.44 nm. Although the calculated grain size is some smaller than that shown in Figure 3a, the CIGS(112) peak should be induced by the CIGS nanoparticles observed by SEM for defects, dislocations, and twins in the grains can lead to smaller calculated grain size than that of the actual one. These crystalline CIGS nanoparticles are beneficial to increase the interface area between the CIGS and P3HT:PCBM blends. In the light absorption spectra (shown in Figure 4a), it could be found that it is these nanoparticles that resulted in the enhancement of the light absorption of the devices.

To investigate the effects of the CIGS nanoparticles on the light absorption and charge separation efficiency of the conjugated polymer active layers, we measured the UV-visible-infrared absorption and PL spectra of the P3HT:PCBM layers with and without the CIGS interlayers (prepared on ITO-glass substrates). Figure 4b displays the absorption spectra of CIGS/ITO, P3HT:PCBM/ITO, sum of CIGS and P3HT:PCBM, and P3HT:PCBM/CIGS/ITO. Obviously, the CIGS interlayer enhances the light absorption of the P3HT:PCBM active layer in the spectral range of 300 to 650 nm. More importantly, the absorption intensity of P3HT:PCBM/CIGS/ITO is much larger than that of the sum of CIGS/ITO and P3HT:PCBM/ITO. It should be noted that the thickness of the P3HT:PCBM monolayer is approximately equal to that of the CIGS/P3HT:PCBM bilayer (about 100 nm) according to the cross-sectional SEM image (see Figure 2c), i.e., the enhancement of light absorption is not due to the thickness change of the P3HT:PCBM layer. Moreover, the CIGS interlayer absorbs only very little incident light. Therefore, most of the increased absorption should come from the P3HT:PCBM close to the interfaces between the P3HT:PCBM and CIGS nanoparticles. The mechanism may be similar to the localized surface plasmon resonant (LSPR) effect [16-20]. It has been known that the excitation of the LSPR through the resonant interaction between the electromagnetic field of incident light and the surface charge of metallic nanostructures causes an electric field enhancement (that can be coupled to the photoactive absorption region) and increases the absorption of photoactive conjugate polymer or organic semiconductor [21-23]. The above results demonstrate that the semiconductor CIGS nanoparticles may also exhibit LSPR effect just as metallic nanostructures do. As demonstrated in Figure 4a, the incident light is trapped by the excitation of localized surface plasmons on the surface of CIGS nanoparticles embedded in P3HT, and the near field of the excited particles causes the creation of the electron-hole pairs in the P3HT. This is very important for the conjugated polymer layers of hybrid solar cells to absorb more incident light (through ITO-glass). If the introduced CIGS interlayer with a narrower bandgap is a continuous thin film rather than scattered nanoparticles, it may absorb too much incident light and decrease rather than increase the light absorption of the photoactive polymer layer behind it. Therefore, the light absorption enhancement induced by the CIGS nanoparticles could permit a considerable reduction in the physical thickness of the conjugated polymer layers in hybrid solar cells and yield some new options for hybrid solar cell design. The PL results in Figure 4c show that the excitons in the polymer are obviously quenched. It has been known that the charge transfer normally occurs with a very high efficiency if excitons are formed in a conducting polymer within approximately 20 nm of a CIGS/P3HT:PCBM interface [23,24]. The above phenomenon suggests that polymer chains were successfully penetrated into the pores of the CIGS nanoparticles, and hole transfer from the polymer to CIGS occurred. The quenching efficiency of a hybrid system can be estimated by calculating the integrated area beneath each curve [25]. The quenching efficiency of P3HT/CIGS in this experiment was calculated to be about 46%.

**Figure 5:**
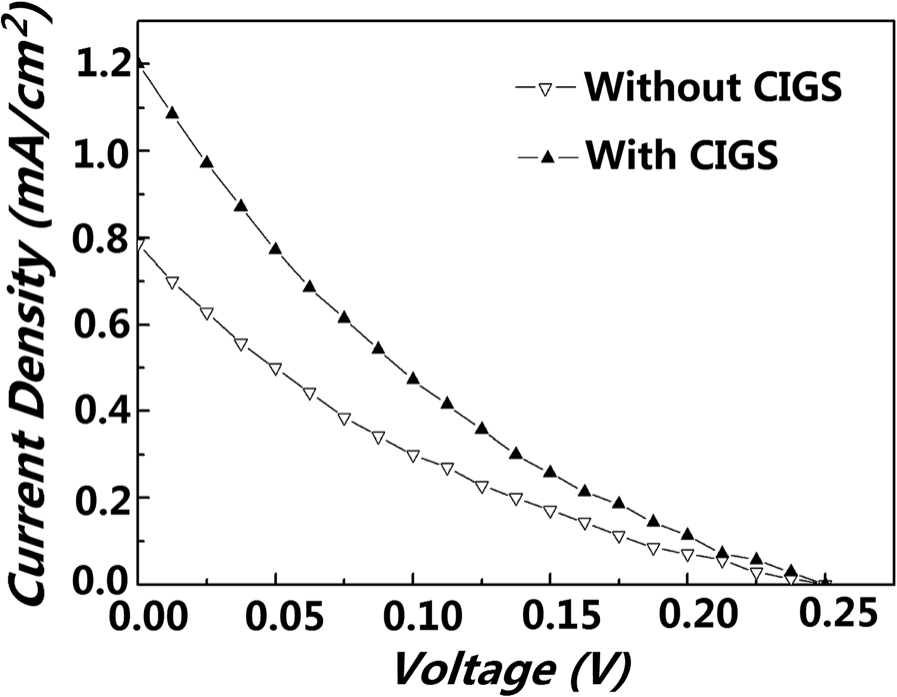
***J*-*V *characteristics.** Comparisons of the *J*-*V* characteristics between the conventional polymer solar cells and hybrid solar cells containing a CIGS interlayer. The photovoltaic properties of the above solar cells were measured under AM 1.5G irradiation at 100 mW/cm^2^.

In order to know the effects of the light absorbance enhancement of the conjugated polymer layer induced by the CIGS nanoparticles on the performance of polymer solar cells, the conventional polymer solar cells (ITO/PEDOT:PSS/P3HT:PCBM/Al) and the hybrid solar cells (ITO/CIGS/P3HT:PCBM/Al) were fabricated, and their *J*-*V* characteristics were tested. The *J*-*V* characteristics of a conventional polymer solar cell and a hybrid solar cell with a CIGS interlayer (as shown in Figure 1) are plotted together in Figure 5 for comparison. The conventional device exhibits a short-current density (*J*_SC_) of 0.77 mA/cm^2^. After introducing a CIGS interlayer deposited by PLD for 3 min (as shown in Figure 2a), the *J*_SC_ increased to 1.20 mA/cm^2^. Since the conventional polymer solar cells and the hybrid solar cells with CIGS interlayers were prepared on almost the same process conditions, these results indicate that the CIGS layers can act as functional interlayers to increase the photocurrents of polymer solar cells. It is hypothesized that the CIGS nanoparticles help the hybrid solar cells produce higher photocurrent by enhancing the light absorption of the conjugated polymer layers. As demonstrated in Figure 1b, the CIGS interlayer (nanoparticles here) with high absorption coefficient (about 10^5^ cm^-1^) itself also absorbs small part of incident light, and the excitons generated in both the P3HT and CIGS nanoparticles separate at the interfaces of both CIGS/PCBM and P3HT/PCBM more efficiently than at the interface of the P3HT/PCBM only in conventional polymer cells. These separated electrons and holes pass through the CIGS layer and polymer layer,respectively. If the CIGS and polymer layers are thin enough, the separated electrons and holes will arrive at the Al cathode and ITO anode with less recombination and larger short-circuit current density.

## Conclusions

The CIGS nanoparticles with sizes of 20 to 70 nm and a distribution density of about 7 × 10^9^ cm^-2^ were deposited on the ITO-glass substrates by PLD. Such CIGS layers were introduced between P3HT:PCBM photoactive layer and ITO-glass substrates to enhance the light absorption of the P3HT:PCBM layer. The UV-visible-infrared absorption and PL spectroscopy measurements of the P3HT:PCBM photoactive layers with and without the CIGS interlayers suggest that the polymer chains are coiled on the CIGS nanoparticles, which enhance the light absorption and improve the efficiency of the exciton separation. The *J*-*V* curves demonstrate that the short-circuit current density of the hybrid solar cells was improved compared with that of the conventional polymer solar cells. These results indicate that the CIGS interlayers composed of nanoparticles are potential to enhance the light absorption of conjugated polymers and improve the photovoltaic performance of polymer solar cells.

## Abbreviations

CIGS: CuIn_0.8_Ga_0.2_Se_2_; EDS: energy-dispersive spectroscopy; LSPR: localized surface plasmon resonant; PL: photoluminescence; PLD: pulsed laser deposition; SEM: scanning electron microscopy; XRD: X-ray diffraction.

## Competing interests

The authors declare that they have no competing interests.

## Authors’ contributions

YZ designed and carried out the experiments and wrote the paper. HL, XL, LG, and YL participated in the experiments. JS, ZY, and JW participated in the design and the discussion of this study. NX conceived and designed the experiments and revised the paper. All authors read and approved the final manuscript.

## Authors’ information

YZ, HL, XL, LG, and YL are graduate students major in fabrication of nanometer materials and optical devices. JS and ZY is an associate professor and MS-degree holder specializing in optics and optical devices. JW is a professor and PhD-degree holder specializing in optics and nanometer materials. NX is a professor and PhD-degree holder specializing in nanometer materials and optical devices, especially expert in nanoscaled optoelectronic devices.

## References

[B1] Yu G Gao J Hummelen JC Wudl F Heeger AJ Polymer photovoltaic cells: enhanced efficiencies via a network of internal donor-acceptor heterojunctions Science 1995 9 5243 1789 1791 10.1126/science.270.5243.1789

[B2] Thompson BC Frechet JMJ Polymer-fullerene composite solar cells Chem IntEd 2008 9 1 58 77 10.1002/anie.200702506 18041798

[B3] Brabec CJ Gowrisanker S Halls JJM Laird D Jia SJ Wliiams SP Polymer-fullerene bulk-heterojunction solar cells Adv Mater 2010 9 34 3839 3856 10.1002/adma.200903697 20717982

[B4] Huynh WU Dittmer JJ Alivisatos AP Hybrid nanorod-polymer solar cells Science 2002 9 5564 2425 2427 10.1126/science.1069156 11923531

[B5] Chandrasekaran J Nithyaprakash D Ajjian KB Maruthamuthu S Manoh Aran D Kumar S Hybrid solar cell based on blending of organic and inorganic materials—an overview Renew Sust Energ Rev 2011 9 2 1228 1236 10.1016/j.rser.2010.09.017

[B6] Bereznev S Konovalov I Opik A Kois J Meuikov E Hybrid copper-indium disulfide/polypyrrole photovoltaic structures prepared by electrodeposition Sol Energ Mat Sol Cell 2005 9 1 197 206

[B7] Arici E Sariciftci NS Meissner D Hybrid solar cells based on nanoparticles of CuInS_2_in organic matrices Adv Funct Mater 2003 9 2 165 171 10.1002/adfm.200390024

[B8] Scharber MC Muhlbacher D Koppe M Denk P Heeger AJ Bra CJ Design rules for donors in bulk-heterojunction solar cells—towards 10% energy-conversion efficiency Adv Mater 2006 9 6 789 794 10.1002/adma.200501717

[B9] Contreras MA Egaas B Ramanathan K Hiltner J Swartzlander A Hasoon F Noufi R Progress toward 20% efficiency in Cu(In, Ga)Se2 polycrystalline thin-film solar cells Prog Photovolt Res Appl 1999 9 4 311 316 10.1002/(SICI)1099-159X(199907/08)7:4<311::AID-PIP274>3.0.CO;2-G

[B10] Song HK Kim SG Kim HJ Kim SK Kang KW Lee JC Yoon KH Preparation of CuIn_1-*x*_Ga_*x*_Se_2_thin films by sputtering and selenization process Sol Energ Mat Sol Cell 2003 9 1–2 145 253

[B11] Kapur VK Bansal A Le P Asensio OI Non-vacuum processing of CuIn_1-*x*_Ga_*x*_Se_2_solar cells on rigid and flexible substrates using nanoparticle precursor inks Thin Solid Films 2003 9 431 432

[B12] Zhang L He Q Jiang WL Liu FF Jiang LC Sun Y Effects of substrate temperature on the structural and electrical properties of Cu(In, Ga)Se_2_ thin films Sol Energ Mat Sol Cell 2009 9 1 114 118 10.1016/j.solmat.2008.09.002

[B13] Levoska J Leppavuori S Wang F Kusmartseva O Hill AE Ahmed E Tomlinson RD Pilkington RD Pulsed laser ablation deposition of CuInSe_2_and CuIn_1-x_Ga_x_Se_2_thin films Phys Scr 1994 9 244 249

[B14] Pavlista M Hrdlicka M Nemec P Prikryl J Frumar M Thickness distribution of thin amorphous chalcogenide films prepared by pulsed laser deposition Appl Phys A 2008 9 3 617 620 10.1007/s00339-008-4687-8

[B15] Huang JS Chou CY Lin CF Enhancing performance of organic–inorganic hybrid solar cells using a fullerene interlayer from all-solution processing Sol Energ Mat Sol Cell 2010 9 2 182 186 10.1016/j.solmat.2009.08.019

[B16] Royer P Goudonnet JP Warmack RJ Ferrell TL Substrate effect on surface plasmon spectra in metal-island films Phys Rev B 1987 9 8 3753 10.1103/PhysRevB.35.3753 9941895

[B17] Barnes WL Dereux A Ebbesen TW Exploitation of localized surface plasmon resonance Nature 2003 9 19 424 824

[B18] Hagglund C Zach M Petersson G Kasemo B Electromagnetic coupling of light into a silicon solar cell by nanodisk plasmons Appl Phys Lett 2008 9 5 053110 10.1063/1.2840676

[B19] Hagglund C Kasemo B Nanoparticle plasmonics for 2D-photovoltaics: mechanisms, optimization, and limits Opt Express 2009 9 14 11944 11957 10.1364/OE.17.011944 19582109

[B20] Harry A Albert P Plasmonics for improved photovoltaic devices Nat Mater 2010 9 10 205 214 20168344 10.1038/nmat2629

[B21] Pei JN Tao JL Zhou YH Dong QF Liu ZY Li ZF Chen FP Zhang JB Xu WQ Tian WJ Efficiency enhancement of polymer solar cells by incorporating a self-assembled layer of silver nanodisks Sol Energ Mat Sol Cell 2011 9 12 3281 3286 10.1016/j.solmat.2011.07.007

[B22] Bellessa J Bonnand C Plenet JC Mugnier J Strong coupling between surface plasmons and excitons in an organic semiconductor Phys Rev Lett 2004 9 3 036404 036408 15323846 10.1103/PhysRevLett.93.036404

[B23] Greenham NC Peng X Alivisatos AP Charge separation and transport in conjugated-polymer/semiconductor-nanocrystal composites studied by photoluminescence quenching and photoconductivity Phys Rev B 1996 9 24 17628 17637 10.1103/PhysRevB.54.17628 9985889

[B24] Hal PA Christiaans MPT Wienk MM Kroon JM Janssen RAJ Photoinduced electron transfer from conjugated polymers to TiO_2_ J Phys Chem B 1999 9 21 4352 4359 10.1021/jp9901803

[B25] Coakley KM Liu Y McGehee MD Frindell KM Stucky GD Infiltrating semiconducting polymers into self-assembled mesoporous titania films for photovoltaic applications Adv Funct Mater 2003 9 4 301 305 10.1002/adfm.200304361

